# The Risk Factors of Acquiring Severe Hand, Foot, and Mouth Disease: A Meta-Analysis

**DOI:** 10.1155/2018/2751457

**Published:** 2018-06-26

**Authors:** Bai Jun Sun, Hui Jie Chen, Ye Chen, Xiang Dong An, Bao Sen Zhou

**Affiliations:** ^1^Department of Epidemiology, China Medical University, Shenyang, Liaoning 110000, China; ^2^Department of Infectious Disease, Shenyang Center for Disease Control and Prevention, Shenyang, Liaoning 110031, China

## Abstract

**Objectives:**

The incidence of severe hand, foot, and mouth disease (HFMD) is not low, especially in mainland China in almost every year recently. In this study, we conducted a meta-analysis to generate large-scale evidence on the risk factors of severe HFMD to provide suggestions on prevention and controlling.

**Methods:**

PubMed, Web of Science, Embase, Cochrane Library, China National Knowledge Infrastructure (CNKI), and Wanfang (Chinese) were searched to identify relevant articles. All analyses were performed using Stata 14.0.

**Results:**

We conducted a meta-analysis of 11 separate studies. Fever (odds ratio (OR) 7.396, 95% confidence interval (CI) 3.565–15.342), fever for more than 3 days (OR 5.773, 95% CI 4.199–7.939), vomiting (OR 6.023, 95% CI 2.598–13.963), limb trembling (OR 42.348, 95% CI 11.765–152.437), dyspnea (OR 12.869, 95% CI 1.948–85.017), contact with HFMD children (OR 5.326, 95% CI 1.263–22.466), rashes on the hips (OR 1.650, 95% CI 1.303–2.090), pathologic reflexes (OR 3057.064, 95% CI 494.409–19000), Lethargy (OR 31.791, 95% CI 3.369–300.020), convulsions (OR 23.652, 95% CI 1.973–283.592), and EV71 infection (OR 9.056, 95% CI 4.102–19.996) were significantly related to the risk of severe HFMD. We did not find an association between female sex (OR 0.918, 95% CI 0.738–1.142), scatter-lived children (OR 1.347, 95% CI 0.245–7.397), floating population (OR 0.847, 95% CI 0.202–3.549), rash on the hands (OR 0.740, 95% CI 0.292–1.874), rash on the foot (OR 0.905, 95% CI 0.645–1.272), the level of the clinic visited first (below the country level) (OR 5.276, 95% CI 0.781–35.630), breast feeding (OR 0.523, 95% CI 0.167–1.643), and the risk of severe HFMD.

**Conclusions:**

Fever, fever for more than 3 days, vomiting, limb trembling, dyspnea, contact with HFMD children, rashes on the hips, pathologic reflexes, lethargy, convulsions, and EV71 infection are risk factors for severe HFMD.

## 1. Introduction

Hand, foot, and mouth disease (HFMD) is a common childhood infection disease with characteristic features of fever, oral ulcers, and vesicular rashes on the hands, feet, and buttocks. It is caused by a group of enteroviruses, commonly coxsackie A16 and enterovirus-71 (EV-71). The mode of transmission of HFMD is mainly via the fecal-oral route, respiratory droplets, contact with blister fluid of an infected individual, or general close contact with infected individuals. Most HFMD cases were mild and limited to fever and vesicular exanthema on patients' palms, soles, and mouth along with discomfortness at certain levels. However, some severe cases with potentially fatal complications such as brain stem encephalitis (BE) and/or pulmonary edema (PE) show rapid progression that may lead to serious sequelae, even death. In recent years, more and more outbreaks of severe cases have been reported [1, 2]. Previous studies have shown that close monitoring and timely intervention may prevent the development of severity and avert the death of severe HFMD [3–5]. Therefore, it is extremely necessary to identify the risk factors which predict the occurrence of severity. Meta-analysis is a means of increasing the effective sample size under investigation through pooling of data from individual association studies, thus enhancing the statistical power [6]. In order to identify risk factors of acquiring severe HFMD, prevent deterioration, and reduce acute mortality, we conducted this meta-analysis to determine the risk factors for severe HFMD.

## 2. Materials and Methods

### 2.1. Study Selection

A systematic search of the literature was done in the following electronic databases: PubMed, Web of Science, Embase, Cochrane Library, China National Knowledge Infrastructure (CNKI), and Wanfang (Chinese). The following keywords were used: severe hand foot mouth disease (HFMD), risk factors, and case control study. The quality of the included studies was assessed using the Newcastle-Ottawa Scale, and studies achieving six or more points were considered to be of high quality.

### 2.2. Inclusion and Exclusion Criteria

Two investigators searched the electronic databases independently according to the following criteria for inclusion: (1) a case-control study including severe and mild disease patient groups; (2) published up to June 2017; (3) diagnosis of severe HFMD and mild HFMD consistent with the criteria defined by us. Abstracts, reviews, case reports, noncomparative studies, and low-quality studies were excluded. In cases of disagreement, a third investigator acted as an arbitrator, and the disagreements were resolved with the research team by discussion.

### 2.3. Data Extraction and Quality Assessment

The following items were extracted from the included studies: the first author's name, year of publication, source of publication, type of the study, risk factors, total sample size, number of severe and mild HFMD cases, and diagnostic criteria for severe and mild HFMD.

The publication bias was evaluated using Egger's test [7]. If *P* > 0.05, the publication bias exists; otherwise, the publication bias does not exist.

### 2.4. Definitions

HFMD cases were divided into two groups according to Guidelines on the Diagnosis and Treatment of HFMD [8]. The mild HFMD was defined as papular/vesicular skin rashes on the hand, foot, mouth, or buttock, and the severe HFMD was defined as mild HFMD with the addition of neurological, respiratory, or circulatory complications, or death. Neurological complications included aseptic meningitis, encephalitis, and acute flaccid paralysis [9]. The duration of fever was defined as body temperature ≥37.5°C.

### 2.5. Meta-Analysis Methods

Stata 14.0 was used for the statistical analysis. The odds ratio (OR) and 95% confidence intervals (CI) were calculated using the fixed effect model or random effect model, and the choice for statistical model was determined by their heterogeneity which were assessed by the *X*^2^ and *I*^2^ statistics. *I*^2^ >50% and *X*^2^-statistic (*P* < 0.1) were considered to show significant heterogeneity, and the random effect model was adopted; otherwise, the fixed effect model was used. The OR and 95% CI were used as summary statistics for the comparison of the following risk factors: fever, fever for more than 3 days, vomiting, limb trembling, dyspnea, contact with HFMD children, rashes on the hips, pathologic reflexes, lethargy, convulsions, EV71 infection, female sex, scatter-lived children, floating population, rash on the hands, rash on the foot, the level of the clinic visited first (below the country level), and breast feeding.

The pooled estimate of risk was obtained by the Mantel–Haenszel method in the fixed effect model and by the M-H heterogeneity method in the random effect model. All *P* values were 2-sided. A *P* value less than 0.05 was considered to be statistically significant.

## 3. Results

### 3.1. Characteristics of Included Studies

**Figure 1:**
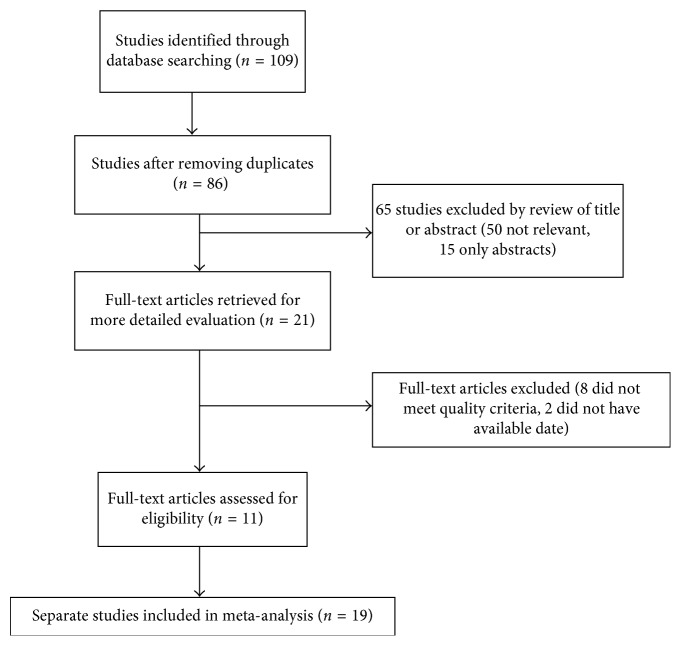
Flow chart showing the selection process for the meta-analysis.

The first search strategy generated 109 studies. Only 11 articles [10–20] met the inclusion criteria, and they were all carried out in China. The selection process is shown in Figure 1. All the studies were of high quality according to the Newcastle-Ottawa Scale (NOS). The sample sizes of the included studies ranged from 76 to 761 and amounted to 4082 subjects in total. There were 1640 patients in the severe HFMD group and 2442 patients in the mild HFMD group. The study and patients' characteristics are summarized in Table 1. The two groups were similar with regard to age and gender.

### 3.2. Risk Factors of Severe HFMD

#### 3.2.1. Patient Clinical Manifestations

**Figure 2:**
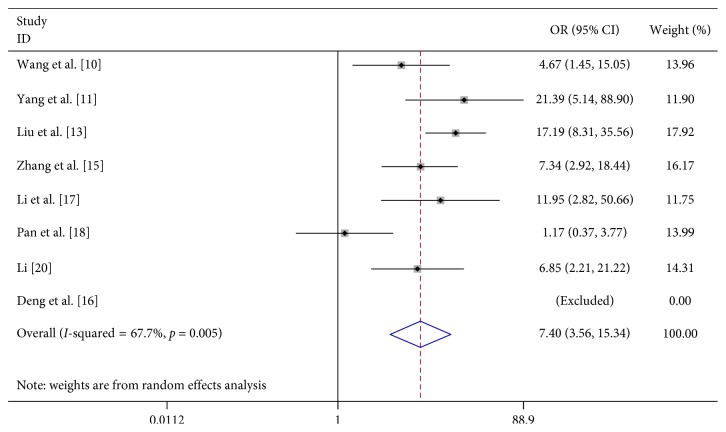
Forest plots showing the results of the meta-analysis regarding fever.

**Figure 3:**
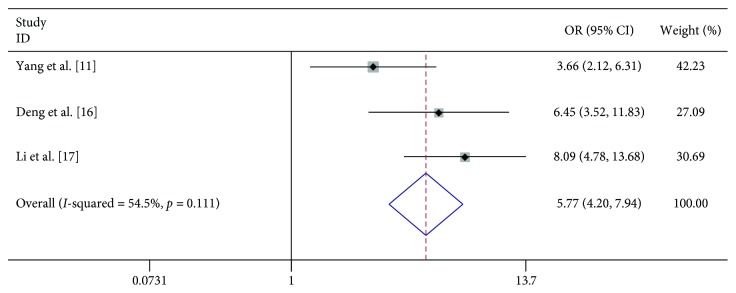
Forest plots showing the results of the meta-analysis regarding fever for more than 3 days.

**Figure 4:**
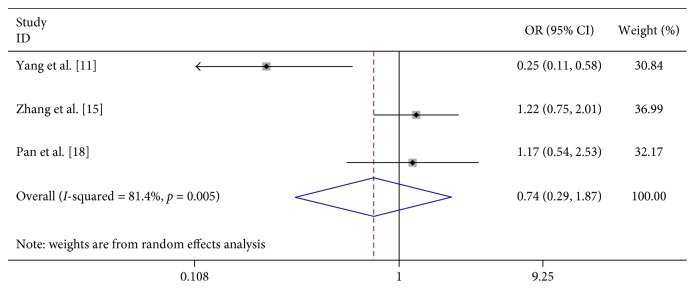
Forest plots showing the results of the meta-analysis regarding rash on the hands.

**Figure 5:**
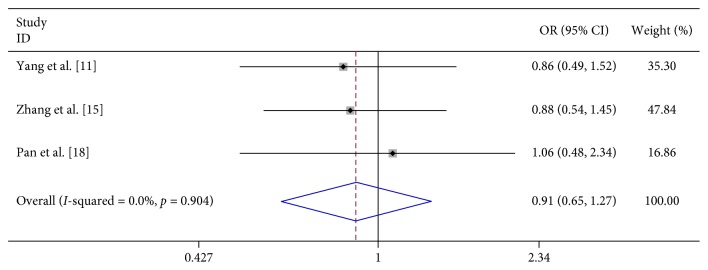
Forest plots showing the results of the meta-analysis regarding rash on the foot.

**Figure 6:**
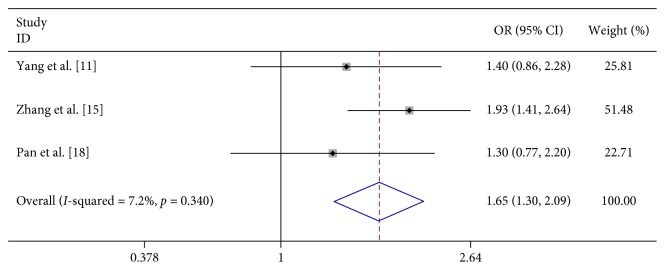
Forest plots showing the results of the meta-analysis regarding rashes on the hips.

In 8 studies, fever was strongly related to the risk of severe HFMD (OR 7.396, 95% CI 3.565–15.342). In 3 studies, fever for more than 3 days was significantly associated with severe HFMD (OR 5.773, 95% CI 4.199–7.939). In 3 studies, rashes on the hips or buttocks were significantly associated with severe HFMD (OR 1.650, 95% CI 1.303–2.090). However, we found no significant association between rash on the palm, rash on the soles, and severe HFMD in 3 studies, respectively (OR 0.740, 95% CI 0.292–1.874 and OR 0.905 95% CI 0.645–1.272) (Table 2; Figures 2–6).

**Figure 7:**
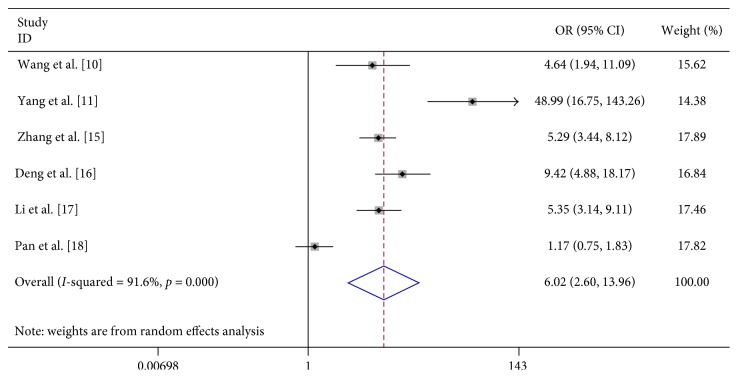
Forest plots showing the results of the meta-analysis regarding vomiting.

**Figure 8:**
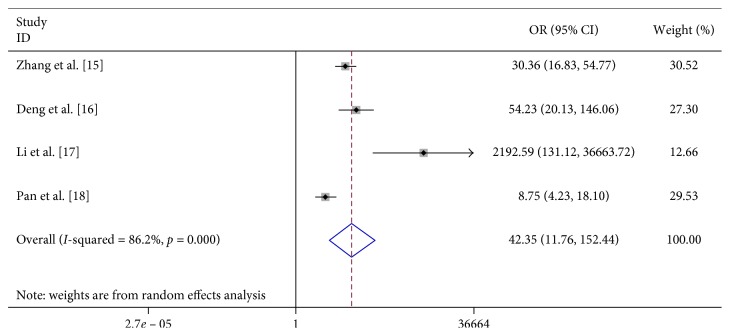
Forest plots showing the results of the meta-analysis regarding limb trembling.

**Figure 9:**
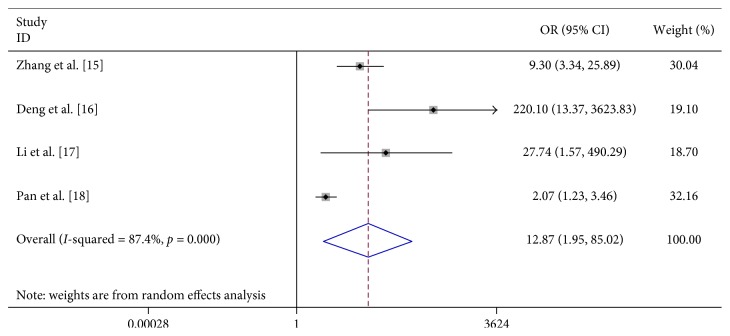
Forest plots showing the results of the meta-analysis regarding dyspnea.

**Figure 10:**
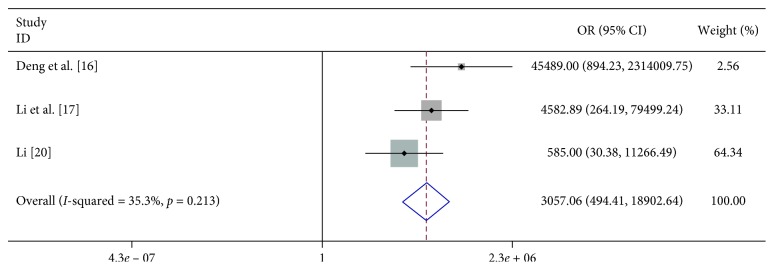
Forest plots showing the results of the meta-analysis regarding pathologic reflexes.

**Figure 11:**
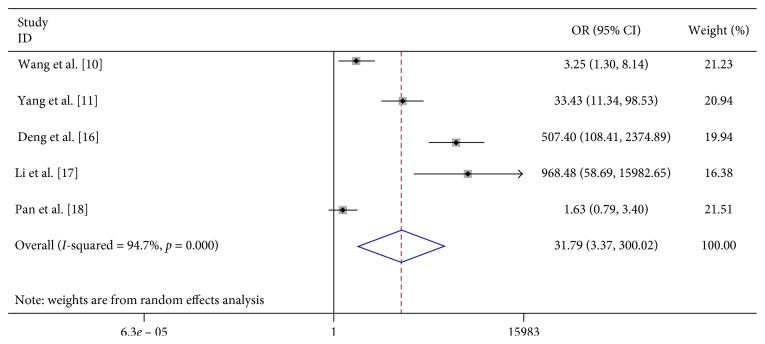
Forest plots showing the results of the meta-analysis regarding lethargy.

**Figure 12:**
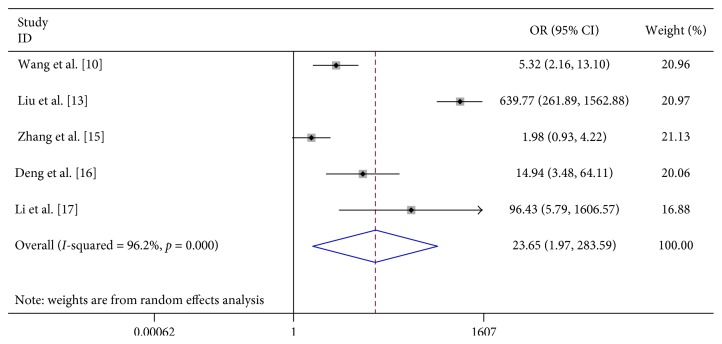
Forest plots showing the results of the meta-analysis regarding convulsions.

We found that vomiting (OR 6.023, 95% CI 2.598–13.963), limb trembling (OR 42.348, 95% CI 11.765–152.437), dyspnea (OR 12.869, 95% CI 1.948–85.017), pathologic reflexes (OR 3057.064, 95% CI 494.409–19000), lethargy (OR 31.791, 95% CI 3.369–300.020), and convulsions (OR 23.652, 95% CI 1.973–283.592) were significantly associated with severe HFMD (Table 2; Figures 7–12).

#### 3.2.2. Patient Demographic Characteristics

**Figure 13:**
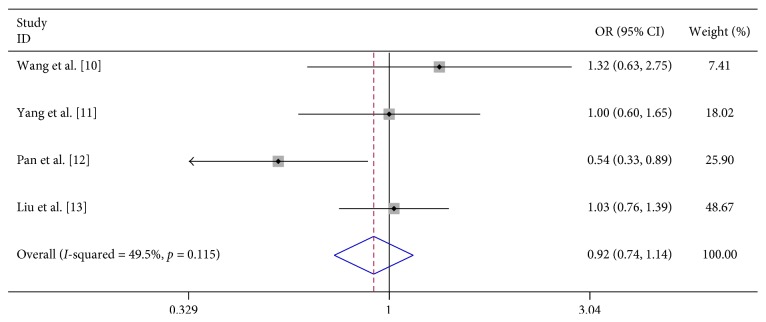
Forest plots showing the results of the meta-analysis regarding female gender.

**Figure 14:**
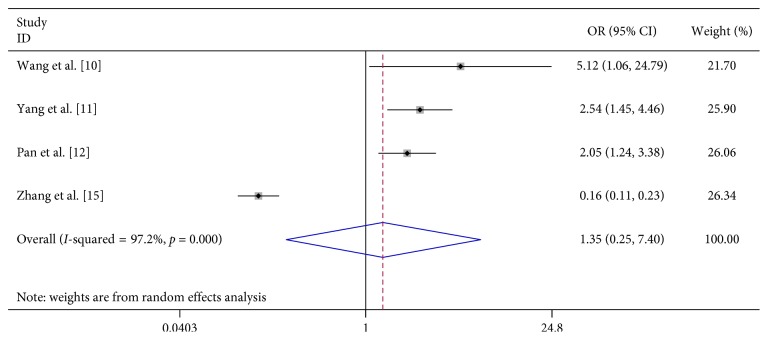
Forest plots showing the results of the meta-analysis regarding scatter-lived children.

**Figure 15:**
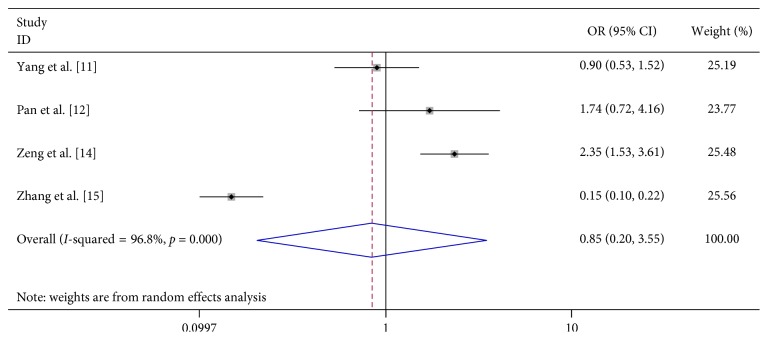
Forest plots showing the results of the meta-analysis regarding floating population.

We analyzed the association between demographic characteristics of patients and severe HFMD and found that female gender (OR 1.06, 95% CI 0.91–1.24), scatter-lived children (OR 1.06, 95% CI 0.91–1.24), and floating population (OR 1.06, 95% CI 0.91–1.24) were all not related to the risk of severe HFMD (Table 2; Figures 13–15).

#### 3.2.3. EV71 Infection

**Figure 16:**
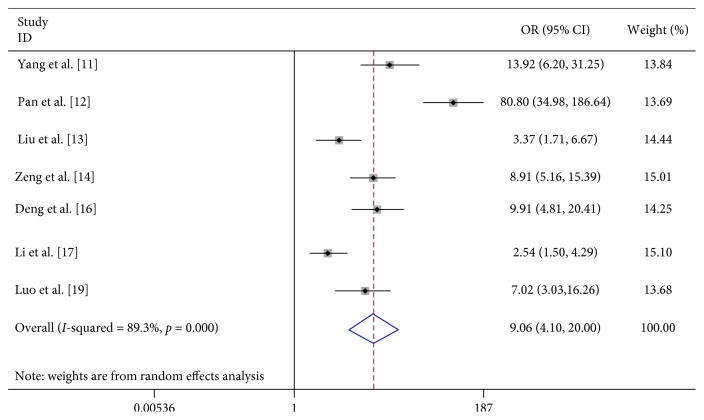
Forest plots showing the results of the meta-analysis regarding EV71 infection.

Seven studies analyzed the association between EV71 infection and severe HFMD, and the results suggest that EV71 infection significantly increased the probability of severe HFMD (OR 9.056, 95% CI 4.102–19.996) (Table 2; Figure 16).

#### 3.2.4. Association between Other Factors and Severe HFMD

**Figure 17:**
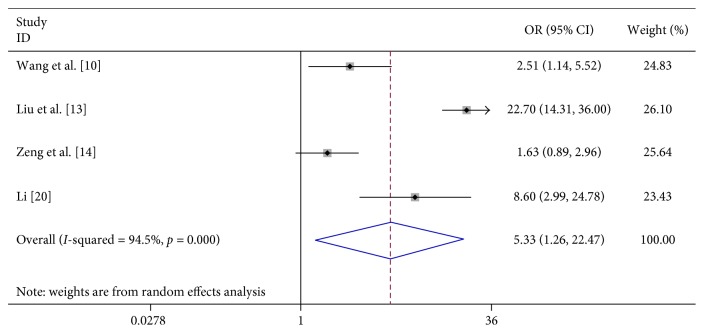
Forest plots showing the results of the meta-analysis regarding contact with HFMD children.

**Figure 18:**
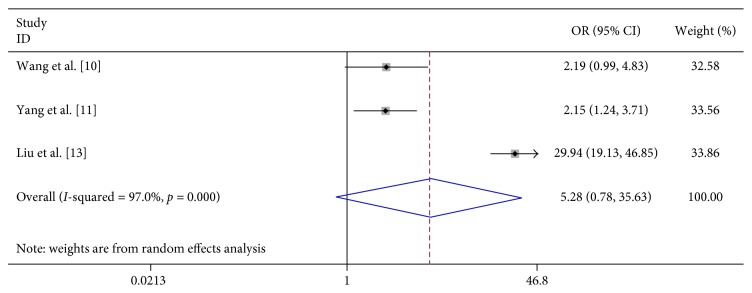
Forest plots showing the results of the meta-analysis regarding the level of the clinic visited first (below the country level).

**Figure 19:**
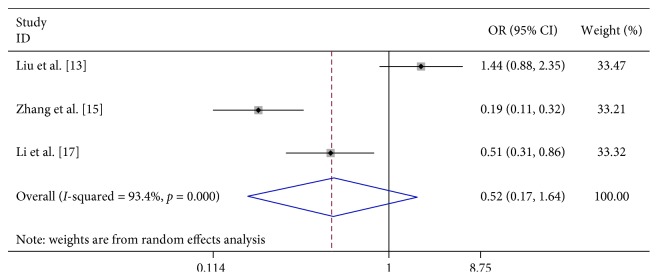
Forest plots showing the results of the meta-analysis regarding breast feeding.

Seven studies suggest that contacting with HFMD children significantly increased the risk of severe HFMD (OR 5.326, 95% CI 1.263–22.466). We found no association between the level of the clinic visited first (below the country level) (OR 5.276, 95% CI 0.781–35.630), breast feeding (OR 0.523, 95% CI 0.167–1.643), and severe HFMD (Table 2; Figures 17–19).

### 3.3. Evaluation of Publication Bias

Egger's test analysis of total complications was performed. The results are shown in Table 3. Seventeen compared factors had no publication bias; one risk factor did (rashes on the hips).

## 4. Discussion

A recent meta-analysis involving 19 separate studies [21] found that clinical characteristics such as duration of fever more than 3 days, body temperature ≥37.5°C, lethargy, vomiting, and EV71 infection were significantly related to the risk of severe HFMD which is consistent with our findings. Other than these risk factors, we also found rashes on the hips or buttocks, limb trembling, dyspnea, pathologic reflexes, convulsions, and contact with HFMD children significantly increased the risk of severe HFMD. However, we found no significant association between rash on the palm, rash on the soles, female gender, scatter-lived children, floating population, the level of the clinic visited first (below the country level), breast feeding, and severe HFMD.

Previous studies on gender have different conclusions: Pan et al. [12] thought that female have a lower risk of attacking severe HFMD than male; however, Wang et al. [10], Yang et al. [11], and Liu et al. [13] do not think that there is a connection, and our analysis found that there is no association between gender and severe HFMD.

Both male and female have the same opportunities to develop severe HFMD. Previous studies have also drawn different conclusions regarding scatter-lived children and floating populations: Zhang et al. [15] thought that scatter-lived children reduce the risk of acquiring severe HFMD, but Wang et al. [10], Yang et al. [11], and Pan et al. [12] got the opposite conclusion. Zhang et al. [15] thought that floating children have a lower risk of acquiring severe HFMD; however, Zeng et al. [14] found a higher risk between them. Yang et al. [11] and Pan et al. [12] did not find any association. Our analysis found both scatter-lived children and floating populations is not related to the risk of severe HFMD.

Oral ulcers and vesicular rashes on the hands, feet, and hip/buttocks are common signs of HFMD; however, rashes on different parts can lead to different levels of severity of HFMD. Previous studies regarding rashes on the hands and rashes on the hips have different conclusions: Yang et al. [11] thought that rashes on the hands are protective factors of HFMD, and other two studies found no association between them; Zhang et al. [15] thought that rashes on the hips are risk factors of HFMD, and other two studies found no association between them. Our studies confirm previous conclusions about the relationship between rashes on the feet and severe HFMD but found there is no association between rashes on the hands and severe HFMD and also found that rashes on the hips are risk factors. These remind us to pay close attention to those kinds of patients whose rashes on the hips.

Most of previous studies included in our meta-analysis found that fever, vomiting, lethargy, convulsions, and contact with HFMD children are risk factors of severe HFMD, and our study confirmed it. Our study also confirmed that severe HFMD is associated with fever for more than 3 days, limb trembling/shaking, dyspnea, pathologic reflexes, and EV71 infection again. Any of these factors increase the possibility of developing severe HFMD. Therefore, early recognition and meticulous management of patients with these risk factors are required [22, 23].

EV71 invades the central nervous system causing severe disease ranging from meningitis to fatal encephalitis [24]. In our study, we retrieved 7 studies that analyzed the association between EV71 infection and severe HFMD, and the meta-analysis showed that EV71 infection was significantly associated with the development of severe HFMD. Enterovirus 71 (EV71) is the key pathogen of HFMD, accounting for 70% severe HFMD cases and 90% HFMD-related deaths [25] Therefore, EV71 vaccine development is very important in preventing severe HFMD epidemics. On December 3, 2015, the China Food and Drug Administration (CFDA) approved the first inactivated enterovirus 71 (EV71) whole virus vaccine for preventing severe hand, foot, and mouth disease (HFMD) [26]. In Mainland China, phase III clinical trials showed that all three EV71 vaccines had good safety and protective efficacy in infants. The protection rates against the EV71-caused HFMD were 97.4%, 94.8%, and 90.0%, respectively, following EV71 vaccination [27–29]. HFMD has become a serious public health issue over the past decades in the Asia-Pacific countries [9]. Inactivated EV71 vaccine will be a valuable tool in protecting children's health in Mainland China and other countries with high HFMD prevalence.

In recent years, coxsackievirus A6 (CV-A6) has caused widespread concern around the world and has gradually emerged as a major pathogen of the hand-foot-mouth disease (HFMD) [30]. The infection caused by CV-A6 is more likely to present with atypical clinical symptoms compared with that by EV-A71 (enterovirus 71, EV-A71) and CV-A16 (coxsackievirus A16, CV-A16) [31], and detoxification is a common clinical manifestation of HFMD caused by CV-A6 [32]. In severe cases, neurological symptoms, aseptic meningitis, and encephalitis occur [33]. However, the current researches on Cox A6 are not comprehensive, and there is a lack of a case-control study on the risk factors of CV-A6. Therefore, more research is needed on coxsackie A6.

Liu et al. [13] reported that the level of the clinic visited first (below the country level) has a marked impact on severe HFMD development. However, our study did not come to the same conclusion. In addition, Zhang et al. [15] found breast feeding is a protective factor. However, our study did not produce the same results also. There may be less to do with the number of studies being included and the less number of samples. Therefore, further studies are needed to be conducted to confirm the association between the level of the clinic visited first and severe HFMD as well as the association between breast feeding and severe HFMD.

## 5. Conclusions

In conclusion, we found that eleven factors are associated with the severity of HFMD. Previous conclusion regarding the association between fever (body temperature ≥37.5°C), fever more than 3 days, lethargy, vomiting, and EV71 infection and severe HFMD was consistent with our findings. Also, we found rashes on the hips or buttocks, limb trembling/shaking, dyspnea/breathlessness, pathologic reflexes, convulsions/twitch, and contact with HFMD children significantly increased the risk of severe HFMD. But, we found no significant association between rashes on the palm, rashes on the soles, female gender, scatter-lived children, floating population/migrant, the level of the clinic visited first (below the country level), breast feeding, and severe HFMD. Further studies are needed to confirm our findings.

## Figures and Tables

**Table 1:** Characteristics of the included studies and patients into meta-analysis.

Author	Year	Country	Severe HFMD	Mild HFMD	Study quality (score)	Age (months)	Female (%)
						Severe HFMD versus mild HFMD	Severe HFMD versus mild HFMD
Wang et al. [10]	2014	China	60	60	∗∗∗∗∗∗	18/17; *P*=NS	43/36; *P*=0.457
Yang et al. [11]	2012	China	89	267	∗∗∗∗∗∗	24/25; *P*=0.345	35/35; *P*=1.000
Pan et al. [12]	2012	China	105	210	∗∗∗∗∗∗	25/32; *P*=NS	30/45; *P*=0.015
Liu et al. [13]	2014	China	249	512	∗∗∗∗∗∗∗	37/37; *P*=NS	45/45; *P* > 0.05
Zeng et al. [14]	2013	China	139	304	∗∗∗∗∗∗	27/25; *P*=NS	35/34; *P*=0.668
Zhang et al. [15]	2017	China	459	246	∗∗∗∗∗∗∗	23/23; *P*=0.356	35/34; *P*=0.976
Deng et al. [16]	2016	China	128	88	∗∗∗∗∗∗	NR	37/39; *P*=0.817
Li et al. [17]	2013	China	116	202	∗∗∗∗∗∗	NR	36/28; *P*=0.137
Pan et al. [18]	2012	China	229	140	∗∗∗∗∗∗	NR	NR
Luo et al. [19]	2016	China	30	373	∗∗∗∗∗∗	NR	NR
Li [20]	2016	China	36	40	∗∗∗∗∗∗	41/39; *P*=NS	47/48; *P* > 0.05

NR: not reported; NS: not significant.Now the Newcastle-Ottawa scale is mainly applied in the evaluation of case-control study. The literature was graded in terms of selection, comparability, and outcome, and each aspect consists of a number of assessment items. When the items are up to the requirements, one star can be obtained, of which the comparability can reach a maximum of 2. Six stars (∗∗∗∗∗∗) and more were considered to be of high quality.

**Table 2:** Meta-analysis of risk factors for the hand, foot, and mouth disease in 18 separate studies.

Risk factors	Number of studies	Severe HFMD		Mild HFMD		OR (95% CI)	Test of heterogeneity			
		Yes	No	Yes	No		Model	Chi-square	*P* value	*I* ^2^ (%)
Female	4	202	301	438	611	0.918 (0.738, 1.142)	F	5.94	0.115	49.5
Scatter-lived/sporadic children	4	370	343	515	268	1.347 (0.245, 7.397)	R	108.61	≤0.001	97.2
Floating population/migrant	4	307	485	377	650	0.847 (0.202, 3.549)	R	94.44	≤0.001	96.8
Fever	8	1334	34	1176	371	7.396 (3.565, 15.342)	R	18.6	0.005	67.7
Fever for more than 3 days	3	207	124	99	370	5.773 (4.199, 7.939)	F	4.39	0.111	54.5
Vomiting	6	478	603	132	870	6.023 (2.598, 13.963)	R	59.18	≤0.001	91.6
Limb trembling/shaking	4	588	343	46	630	42.348 (11.765, 152.437)	R	21.76	≤0.001	86.2
Dyspnea/breathlessness	4	210	721	29	647	12.869 (1.948, 85.017)	R	23.81	≤0.001	87.4
Contact with HFMD children	4	215	269	84	832	5.326 (1.263, 22.466)	R	54.58	≤0.001	94.5
Rash on the palm/hands	3	700	75	601	52	0.740 (0.292, 1.874)	R	10.73	0.005	81.4
Rash on the soles/foot	3	680	92	558	93	0.905 (0.645, 1.272)	F	0.2	0.904	0
Rashes on the hips/buttocks	3	533	237	368	285	1.650 (1.303, 2.090)	F	2.16	0.340	7.2
Level of the clinic visited first (below the country level)	3	238	160	114	725	5.276 (0.781, 35.630)	R	65.87	≤0.001	97
Breast feeding	3	613	196	824	129	0.523 (0.167, 1.643)	R	30.46	≤0.001	93.4
Pathologic reflexes	3	267	13	0	330	3057.064 (494.409, 19000)	F	3.09	0.213	35.3
Lethargy/hypersomnia	5	278	344	25	732	31.791 (3.369, 300.020)	R	76.16	≤0.001	94.7
Convulsions/twitch	5	334	676	25	1083	23.652 (1.973, 283.592)	R	104.46	≤0.001	96.2
EV71 infection	7	521	149	586	1012	9.056 (4.102, 19.996)	R	56.03	≤0.001	89.3

OR, odds ratio; CI, confidence interval; HFMD, hand, foot, and mouth disease; EV71, enterovirus 71; R, random effect model; F, fixed effect model.

**Table 3:** Egger's test of all risk factors.

Risk factors	Number of studies	Egger's test		Publication bias (yes or no)	Model
		*t* value	*P* value		
Female	4	−0.12	0.916	No	F
Scatter-lived children	4	1.18	0.361	No	R
Floating population	4	0.58	0.620	No	R
Fever	8	−0.74	0.492	No	R
Fever for more than 3 days	3	−0.04	0.976	No	F
Vomiting	6	1.68	0.168	No	R
Limb trembling	4	1.36	0.306	No	R
Dyspnea	4	3.51	0.072	No	R
Contact with HFMD children	4	−0.72	0.548	No	R
Rash on the hands	3	−1.00	0.499	No	R
Rash on the foot	3	1.92	0.306	No	F
Rashes on the hips	3	−84.52	0.008	Yes	F
Level of the clinic visited first (below the country level)	3	−1.10	0.470	No	R
Breast feeding	3	−9.69	0.065	No	R
Pathologic reflexes	3	1.18	0.448	No	F
Lethargy	5	3.09	0.054	No	R
Convulsions	5	0.40	0.718	No	R
EV71 infection	7	1.83	0.126	No	R

R, random effect model; F, fixed effect model.
